# Sex-Based Medicine Meets Psoriatic Arthritis: Lessons Learned and to Learn

**DOI:** 10.3389/fimmu.2022.849560

**Published:** 2022-04-22

**Authors:** Nicola Luigi Bragazzi, Charlie Bridgewood, Abdulla Watad, Giovanni Damiani, Dennis McGonagle

**Affiliations:** ^1^ Laboratory for Industrial and Applied Mathematics (LIAM), Department of Mathematics, York University, Toronto, ON, Canada; ^2^ Postgraduate School of Public Health, Department of Health Sciences (DISSAL), University of Genoa, Genoa, Italy; ^3^ Leeds Institute of Rheumatic and Musculoskeletal Medicine, University of Leeds, Leeds, United Kingdom; ^4^ Department of Medicine B, Rheumatology Unit and Zabludowicz Center for Autoimmune Diseases, Sheba Medical Center, Ramat-Gan, Israel; ^5^ Sackler Faculty of Medicine, Tel-Aviv University, Tel-Aviv, Israel; ^6^ Clinical Dermatology, Istituto di Ricovero e Cura a Carattere Scientifico (IRCCS) Galeazzi Orthopaedic Institute, Milan, Italy; ^7^ National Institute for Health Research Leeds Biomedical Research Centre, Leeds Teaching Hospitals, Leeds, United Kingdom

**Keywords:** sex-based medicine, sex-specific differences, psoriatic arthritis, epidemiology, clinical presentation, radiological and laboratory features, response to treatment

## Abstract

Humorally associated autoimmune diseases generally show a female predominance whereas ankylosing spondylitis, a disease that overlaps with psoriatic arthritis (PsA), shows a male predominance. The present review ascertains the current knowledge of sex-specific differences related to psoriatic arthritis (PsA), a chronic, inflammatory condition associated with psoriasis. Sex differences may have important implications for clinical research in PsA and in terms of epidemiology (incidence, prevalence, lifetime risk, survival, and mortality), clinical, radiological, and laboratory features, and response to treatment. While nationwide surveys and large-scale databases and registries show no sex-specific differences, varying male/female ratios have been reported, ranging from 0.42 to 2.75 (comparable with those reported for psoriasis vulgaris: ranging from 0.28 to 2.38). This may reflect subtle, complex, nonlinear interactions between the biological make-up of the individual (genetic and epigenetic differences), hormonal components including menopausal status, environmental exposures including skeletal physical stressing, and psychological variables. There exists methodological heterogeneity and paucity of data concerning sex-specific differences, in terms of the specific population studied, study design, and the diagnostic criteria utilized. Harmonizing and reconciling these discrepancies would be of crucial importance in achieving the ambitious goals of personalized/individualized medicine and further standardized meta-data and Big Data could help disentangle and elucidate the precise mechanisms of underlying potential PsA sex-specific differences.

## Sex-Based Medicine

Sex variables and their subtle, complex, and non-linear interactions can dramatically influence health-related outcomes and may profoundly impact autoimmune, inflammatory, and infectious diseases (1). The still ongoing “Coronavirus Disease 2019” (COVID-19) pandemic has shown how adverse prognosis depends on male sex and how it is of paramount importance (2, 3).

As stated in 2001 and 2010 by the USA “National Academy of Medicine”, previously known as “Institute of Medicine” (IoM), and in 2010 by the Canadian Institutes of Health, these variables are, indeed, not related/restricted to reproductive and sexual health and wellbeing only, but they encompass the entire human organism as a whole, from a biological, psychological, and societal perspective, deeply influencing its perceived health-related quality of life. Moreover, implementing approaches that integrate sex-related aspects would impact biomedical practices, including the delivery of healthcare provisions, enabling the ambitious achievements of the so-called personalized/individualized medicine, in which the needs of each subject are considered, instead of a “one-size-fits-all” approach.

Sex-specific differences profoundly influence the natural history of a disease, its etiopathogenesis, and the biological mechanisms underlying its insurgence and progression as well as its diagnosis, prognosis, outcomes, response to treatment, and preventative strategies (4). Despite this, sex-specific medicine is still neglected (4), with information concerning sex-related biomedical aspects being overlooked or not consistently incorporated in the medical and allied health profession undergraduate, graduate, and post-graduate training and curricula. Concerning COVID-19, a recently published study found that, even though sex has implications for COVID-19 vaccine effectiveness and safety profile, out of 75 interventional and observational COVID-19 vaccines trials, only 24% presented endpoint outcomes stratified by sex, and only 13% discussed the implication of this (5).

In the field of rheumatology, sex-specific differences have consistently been reported for various diseases, including rheumatoid arthritis (6, 7), axial spondyloarthritis (8), or ankylosing spondylitis (9). Despite this, bias based on sex persists in clinical rheumatology.

All of this emphasizes the importance of integrating sex-based medicine into clinical practice and research. Sex-based medicine can be defined as “a study of the differences in men’s and women’s normal function and in their experience of the same diseases”, according to the classical definition by Marianne Legato (10).

## Psoriatic Arthritis

Psoriatic arthritis (PsA) is a chronic inflammatory condition associated with cutaneous psoriasis, and it can be conceived as a wide clinical phenotype, with some proposed differences between sexes; however, these have not been investigated systematically and fail to be incorporated in both research and daily clinical practice despite their relevance.

According to a recent study conducted according to the “Global Burden of Disease” (GBD) 2019 methodology (11), there were more than 4.6 million incident cases of psoriasis worldwide in 2019, with an age-standardized incidence rate of 57.8 per 100,000 people. Compared to 1990, this corresponded to a reduction of 20.0%. By sex, the age-standardized incidence rate was comparable between males and females. Compared to 1990, this corresponded to a reduction of 19.5% and 20.4%, respectively. The age-standardized incidence rate per 100,000 persons widely varied across geographic settings, with high-income countries and territories reporting the highest rate of psoriasis, followed by high-middle income countries. Similar patterns could be found for the other GBD-related metrics, including prevalence and years of life with a disability (YLDs).

Up to approximately 30% of psoriatic patients develop PsA, which can result in irreversible joint damage. According to a recently published systematic review of the literature and meta-analysis (12), the degree of psoriasis severity and nail pitting were found to be predictors of early-onset PsA insurgence and development, as were a higher body mass index (BMI) and a family history of PsA. Psoriatic patients with arthralgia (overall relative risk or RR 2.15 [95% confidence interval or CI ranging from 1.16 to 3.99]) and/or with imaging-musculoskeletal inflammation (pooled RR 3.72 [95%CI 2.12 to 6.51]) were deemed at higher risk for developing PsA.

## Etiopathogenetic and Genetic/Post-Genetic Sex-Specific Differences

Sexual dimorphism concerning etiopathogenesis and genetic factors involved in the etiology of psoriatic arthritis is inconsistently reported in the literature.

A pioneering study by Gladman et al. (13) could not find any sex-specific difference in terms of HLA-B*27 or any other psoriatic arthritis-related HLA antigen frequency in a sample of 194 patients with PsA axial disease (82 females, 112 males). These results were partly replicated in a subsequent study (14), assessing 590 psoriatic patients. The authors found that the frequency of HLA-B*27 was comparable among males and females (16.8% versus 15.2% in males and females, respectively), whilst the HLA-C*06 allele occurred more frequently among female patients (33.2% versus 21.9%, statistically significant at p=0.005).

On the other hand, a study by Queiro et al. (15) found that the frequency of HLA-B*27 was higher among male patients with psoriatic spondyloarthritis compared to their female counterparts (48% versus 11%, statistically significant at p=0.002), whereas no difference could be found in terms of the frequency of HLA-C*06 (54% versus 58% in males and in females, respectively). The cohort analyzed consisted of 100 patients (63 male and 37 female).

These discrepancies could be explained by taking into account different samples, various countries (and putative ethnic factors), as well as sampling techniques. On the other hand, as previously mentioned, psoriatic arthritis is a wide clinical phenotype and the statistical significance (or the lack of statistical significance) of certain alleles could reflect certain endotypes/sub-phenotypes (16).

Furthermore, Rahman et al. (17) reported data suggestive of the presence of genomic imprinting or a similar phenomenon among psoriatic arthritis patients, with the proportion of patients with an affected father being significantly higher than the expected computed proportion (0.65 vs 0.50, statistically significant at p=0.001).

The “imprinting hypothesis” for psoriatic arthritis has been confirmed by a linkage scan study in 178 patients (18), which was able to dissect a genetic component characterized by segregation within families and high concordance among identical twins. A linkage was found with a “logarithm of the odds” (LOD) score of 2.17 on chromosome 16q. The score was computed at 4.19 when conditioned for paternal transmission and 1.03 when conditioned for maternal transmission.

Finally, Hong et al. (19) performed a two-sample, sex-stratified Mendelian randomization analysis utilizing datasets from genome-wide association studies. IL-6 was associated with psoriatic arthritis in the male population but not in the female population.

## Epidemiological Sex-Specific Differences

As for psoriasis, the epidemiology of psoriatic arthritis does not generally differ between males and females (11, 20) if we consider nationwide surveys and large-scale databases and registries. However, some studies have reported varying male/female ratios, ranging from 0.42 to 2.75 (20–29) (**Table 1**). For instance, Andersen and Davis (21), utilizing Rochester Epidemiology Project data, found a higher rate of psoriatic arthritis among males than among females. These findings replicated those by Shbeeb et al. (22) and by Wilson et al. (23). Similarly, recently, Karmacharya et al. (24) conducted a population-based cohort study based in Olmsted County, Minnesota, USA, in the period from 1970 to 2017. In the sub-period 2000-2017, the overall age- and sex-adjusted annual incidence of psoriatic arthritis per 100,000 population was only slightly higher among males than among females (9.3 [95% confidence interval, CI, 7.4-11.3] for males, 7.7 [95%CI 5.9-9.4] for females). The incidence rate was stable, with a 3% yearly increase among females.

**Table 1:** An overview of the major studies/surveys reporting sex-stratified epidemiological rates of both psoriatic arthritis and psoriasis.

Study	Male/female ratio	Country/countries
**Psoriatic arthritis**		
Hoff et al. (27)	0.89	Norway
Karmacharya et al. (24)	1.21	USA
Nossent and Gran (25)	1.52	Norway
Savolainen et al. (28)	0.68	Finland
Scotti et al. (20)	1.13	Overall; Sweden (0.42-1.05), The Netherlands (NC), Finland (0.68-1.3), Greece (0.50), USA (0.65-1.58), Norway (0.96-1.17), Italy (NC), France (0.50), Iceland (0.70), Lithuania (1.25), Czech Republic (1.3), Mexico (NC), Argentina (2.49), Turkey (0.65), China (0.0), UK (NC)
Shbeeb et al. (22)	1.00	USA
Soderlin et al. (29)	0.42	Sweden
Soriano et al. (26)	2.75	Argentina
Wilson et al. (23)	1.69	USA
**Psoriasis vulgaris**		
Bell et al. (30)	From 0.31 to 2.38	USA
Egeberg et al. (31, 32)	From 0.81 to 1.01	Denmark
Huerta et al. (33)	From 0.72 to 2.11	UK
Icen et al. (34)	From 0.94 to 2.01	USA
Khalid et al. (35)	From 0.71 to 1.20	UK
Pezzolo et al. (36)	From 0.28 to 1.73	Italy
Schonmann et al. (37)	From 1.04 to 1.12	Israel
Sewerin et al. (38)	From 0.75 to 1.00	Germany
Springate et al. (55)	From 0.91 to 1.02	UK
Tillett et al. (39)	From 0.81 to 1.09	UK
Tollefson et al. (40)	From 0.60 to 1.10	USA
Vena et al. (41)	1.23	Italy

NC, not computable.

Nossent and Gran (25) reported a male-to-female ratio of 1.52 in their study based in Norway, whilst Soriano et al. (26) reported an even higher ratio of 2.75 in a survey from Argentina. Ratios below 1, indicating a rate slightly higher among females compared to males, were computed by Hoff et al. (27) from Norway, by Savoilanen et al. (28) from Finland, and by Soderlin et al. (29) from Sweden.

According to Crowson et al. (42), the lifetime risk of adult-onset psoriatic arthritis was computed to be 0.46 [95%CI 0.33-0.59] for females and 0.62 [95%CI 0.51-0.72] for males, being slightly higher for the latter.

Such discrepancies may depend on the specific population studied, the methodology adopted, including the study design and the nature of the investigation (single- versus multi-center), a reflection of probable selection/sampling bias, as well as the lack of internationally validated/standardized criteria for case identification and classification of the disease (11, 20, 43). Several criteria and operational definitions, indeed, exist, including the Vasey and Espinoza method (44), the methods of Taylor and Helliwell (45, 46), McGonagle et al. (47), Bennett (48), Moll and Wright (49), Gladman et al. (50), Fournie et al. (51), the “European Spondyloarthropathy Study Group” (ESSG) (52), and the “ClASsification criteria for Psoriatic Arthritis” (CASPAR) (53), reviewed in (54–56).

Moreover, in most cases data were crude/unadjusted, with sex-specific data standardization being provided in a few cases. Furthermore, discrepancies in the epidemiological rates may reflect the impact of ethnicity, but this aspect is relatively overlooked with most studies being conducted in Europe and North America, and, as such, it warrants further investigation.

Considering psoriasis, a recently published systematic review (30–41, 57, 58) reported contrasting findings related to sex-specific male/female rates, ranging from 0.28 to 2.38, with the disease showing a bimodal presentation and presenting slightly earlier in females compared to males. In children, the incidence rate was lower in males than females (computed male/female ratio of 0.86). Among adults, results were inconsistent across studies and over time. No differences in terms of prevalence rate could be reported in studies based in Brazil and Taiwan, which is different compared to investigations conducted in Denmark, Germany, and Sweden, which found a prevalence rate slightly higher rate among females when compared to males. A study from China, sampling from adolescent populations, computed a rate five times higher among females. When considering all age groups, most studies reported a prevalence rate slightly higher among males, with the exception of investigations conducted in Denmark, Norway, Poland, Scotland, and Sweden. Interestingly, a study based in Australia computed a rate that was double among males compared to females.

On the other hand, if the incidence and prevalence rates of psoriatic arthritis have been intensively studied, less is known about imposed mortality. In the study by Karmacharya et al. (24), no sex-specific differences could be found for overall survival and standardized mortality ratio. However, Elalouf et al. (59) reported different findings for survival and mortality in a cohort of 1,490 patients followed over 15062.8 patient-years. The authors computed 225 (15%) confirmed deaths (111 among females, and 114 among males). The overall standardized mortality ratio was computed at 0.92 [95%CI 0.81-1.05]. When stratifying according to sex, the sex-specific standardized mortality ratio was slightly higher among females than among males (1.08 [95%CI 0.89-1.30] for females, 0.81 [95%CI 0.66-0.97] for males). Fagerli et al. (60) reported conflicting results. The authors linked 709 psoriatic patients under biologics included in the British Society for Rheumatology Biologics Register with the UK national cancer and the national death registers, totaling 5,286 patient-years of follow-up. Incidence of nonmelanoma skin cancer was significantly higher among females, with respect to the general population, while, among males, did not differ from the general population. Males had a 75% increased mortality rate (with a standardized mortality ratio of 1.75 [95%CI 1.11-2.63]) with respect to the general population, differently from females. Similarly, rates for death from circulatory disease, and specifically for coronary heart disease, were significantly higher among males.

## Clinical, Radiological, and Laboratory Sex-Specific Differences

In the study by Queiro et al. (15), 23 patients exhibited isolated axial disease (with a male-to-female ratio of 3.6:1), 41 showed an oligoaxial pattern (with a male-to-female ratio of 1.7:1), while 36 had a polyaxial disease (with no differences between males and females, with a male-to-female ratio of 1:1). Interestingly, the frequency of HLA-B*27 correlated with isolated axial disease (p=0.016). Moreover, being female was associated with lower complement levels (with a p-value less than 0.05), erosive and aggressive disease (p=0.05), higher swelling joint count (p=0.002), poorer functional outcomes, and higher scores on the Health Assessment Questionnaire-Specific for spondyloarthropathy (with a p-value less than 0.05).

Duruöz (61) assessed a cohort of 1,038 psoriatic arthritis patients (678 females and 360 males) and found that females showed higher disease activity scores, in terms of “Disease Activity Score 28” (DAS28) and “clinical Disease Activity Index for Psoriatic Arthritis” (cDAPSA), with males reporting higher remission rates. “Minimal Disease Activity” (MDA) exhibited sex-specific differences too, favoring male patients. However, no differences could be reported for “very low disease activity” (VLDA). The incidence rates of enthesitis, dactylitis, tenosynovitis, and inflammatory bowel disease (IBD) were comparable between males and females, with the exception of spondylitis, which was found to be higher among males. Furthermore, females reported higher BMI and late-onset disease, as well as higher “Hospital Anxiety and Depression Scale” (HAD), “Health Assessment Questionnaire” (HAQ), and “Fibromyalgia Rapid Screening Tool” (FiRST) scores. Short-Form 36 (SF-36) scores were lower than those reported by male patients.

Similarly, Eder et al. (14) performed a cross-sectional analysis of 590 psoriatic patients (345 males and 245 females) and found that axial involvement was more common among male patients (42.9% versus 31%, statistically significant at p=0.003). This was true also for the multivariate regression analysis, accounting for potential confounding parameters (odds ratio, OR, of 1.8, statistically significant at p=0.003). Moreover, males had a higher likelihood of developing a more severe and aggressive course of the disease, with radiographic damage affecting peripheral joints (OR 1.6, statistically significant at p=0.007). On the other hand, females reported lower functional outcomes and complained of lower quality of life.

These findings were replicated by a study by Queiro et al. (62), who analyzed a cohort of 110 patients and reported males with spondyloarthritis suffering from more severe spinal disease and females having peripheral joint involvement (40% versus 20%, with a p-value less than 0.05).

Nas et al. (63) assessed a cohort of 187 psoriatic arthritis patients (115 females and 72 males) and found that females had higher symptom duration and BMI, tender and swollen joint counts, DAS28, erythrocyte sedimentation rate (ESR), fatigue and unhealthy lifestyles (poorer physical activity practice). The “Psoriasis Area and Severity Index” (PASI) score was higher in male patients. Other features (namely, SF-36 and articular and extra-articular patterns, such as uveitis or iritis), as well as family history of psoriasis, spondyloarthritis, and ankylosing spondylitis, were comparable between male and female patients.

Recently, Kojanova et al. (64) mined BIOREP, a Czech registry of psoriatic patients under biologics. Females had a higher odds of being diagnosed with psoriatic arthritis (43.5% versus 33.0%, with a p-value less than 0.0012). The frequency of comorbidities was comparable between males and females, with the exception of depression, which affected more females than males (11.4% females versus 3.7% males, statistically significant with a p-value less than 0.0012). The “Dermatology Life Quality Index” (DLQI) and the PASI scores were significantly higher and lower in females, respectively (17.6 versus 16.0 for DLQI, 17.7 versus 19.5 for PASI, both statistically significant with a p-value less than 0.0012).

## Sex-Specific Differences Related to Treatment

The treatment for PsA is based on traditional disease-modifying anti-rheumatic drugs (DMARD) and biological agents. Clinical trials have sometimes reported various outcomes in terms of response patterns based on sex. Generali et al. (65) performed a systematic review of the literature, with the aim of identifying sex differences underlying drug retention rate in PsA patients. The authors were able to include and synthesize nine studies, with only one focusing on DMARDs and the other eight investigations concerning biologics targeting the anti-tumor necrosis factor (TNF), including etanercept, adalimumab, and infliximab. While no sex-specific differences could be identified in terms of retention rate and response pattern to methotrexate, female patients exhibited a lower retention rate than their male counterparts with respect to anti-TNF biologics.

In the study by Kojanova et al. (64), females were significantly older than males at the onset of the treatment, with a longer mean duration of the disease and a longer waiting time between diagnosis and commencement of the pharmacological treatment. Moreover, the survival probability under biologics was significantly lower in females for both biologically naïve and non-naïve patients. Furthermore, the occurrence of adverse events was higher among females.

Carvalho et al. (66) collected data about 180 polyarticular psoriatic arthritis patients under TNF blocker from the Rheumatic Diseases Portuguese Register (54% female) and found that, at 3 months, female patients were less likely to achieve a good/moderate EULAR response (OR from 0.082 [95%CI 0.024-0.278] to 0.091 [95%CI 0.011-0.091]) and remission (OR 0.083 [95%CI 0.017-0.416]).

## Psoriatic Arthritis and Sex-Specific Differences

There is a dearth of information concerning the impact of sex-related issues on PsA, the epidemiological trends of which among sex-diverse populations have been and are still overlooked in the literature. Little is known about the effects of endocrine hormones on the development of PsA (67) and even less is known about the impact of hormone replacement therapy among transexual individuals (68, 69). More studies need to focus on these aspects.

## Conclusions

Sex-specific differences have important implications for clinical research and practice. However, they are particularly overlooked in several biomedical disciplines, including the rheumatological arena. Contrasting findings have been reported for PsA. This may reflect subtle, complex, nonlinear interactions between the biological make-up of the individual (genetic and post-genetic complements), hormonal components, environmental exposures including biophysical stress, and psychological variables. On the other hand, this reflects also the heterogeneity and paucity of data concerning sex-specific differences, which would be of crucial importance in achieving the ambitious goals of personalized/individualized medicine. Collaborative networking, international registries, standardized meta-data, and Big Data could help disentangle and elucidate the precise mechanisms underlying sex-specific differences in psoriatic arthritis and advance a customized treatment and management, identifying specific psoriatic arthritis endotypes/sub-phenotypes. The current gaps in knowledge and the methodological shortcomings warrant further high-quality studies.

## Author Contributions

NB conceived and drafted the paper. All other authors critically revised the manuscript. All authors contributed to the article and approved the submitted version.

## Funding

NLB was partially funded by the Celgene supported PARTNER fellowship programme.

## Conflict of Interest

The authors declare that the research was conducted in the absence of any commercial or financial relationships that could be construed as a potential conflict of interest.

## Publisher’s Note

All claims expressed in this article are solely those of the authors and do not necessarily represent those of their affiliated organizations, or those of the publisher, the editors and the reviewers. Any product that may be evaluated in this article, or claim that may be made by its manufacturer, is not guaranteed or endorsed by the publisher.
